# Food Allergy Test‐Guided Dietary Advice for Children With Atopic Dermatitis: A Consensus Study

**DOI:** 10.1111/pde.15807

**Published:** 2024-11-11

**Authors:** Ludivine Garside, Robert Boyle, Rosan Meyer, Isabel Skypala, Hilary Allen, Paula Beattie, Justine Dempsey, Matt Doyle, Helen Evans‐Howells, Mary Feeney, Siân Ludman, Tom Marrs, Jane Ravenscroft, Gary Stiefel, Thisanayagam Umasunthar, Deepan Vyas, Natalie Yerlett, Jo Walsh, Sara J. Brown, Matthew J. Ridd

**Affiliations:** ^1^ Centre for Applied Excellence in Skin & Allergy Research University of Bristol Bristol UK; ^2^ National Heart and Lung Institute Imperial College London London UK; ^3^ Winchester University Winchester UK; ^4^ KU Leuven Leuven Belgium; ^5^ Guy's and St Thomas' NHS Foundation Trust London UK; ^6^ Imperial College London London UK; ^7^ Royal Hospital for Children Glasgow UK; ^8^ Imperial College Healthcare NHS Trust London UK; ^9^ Jersey Allergy Clinic St Lawrence Jersey UK; ^10^ Dr Helen Allergy Bournemouth UK; ^11^ King's College London London UK; ^12^ Royal Devon University Healthcare NHS Foundation Trust Exeter UK; ^13^ Nottingham University Hospitals NHS Trust Nottingham UK; ^14^ University Hospitals of Leicester NHS Trust Leicester UK; ^15^ Oxford University Hospitals NHS Foundation Trust Oxford UK; ^16^ West Hertfordshire Hospitals NHS Trust Watford UK; ^17^ Great Ormond Street Hospital for Children NHS Foundation Trust London UK; ^18^ Castle Partnership Norwich UK; ^19^ University of Edinburgh Edinburgh UK

**Keywords:** atopic dermatitis, consensus, diet therapy, food hypersensitivity, skin tests

## Abstract

**Background:**

The use of blood specific IgE or skin prick tests (SPT) to guide dietary exclusions for disease control in children with atopic dermatitis (AD) is controversial. We undertook a consensus exercise on how to interpret SPT results and dietary history for cow's milk, hen's egg, wheat, and soy in children < 2 years old with AD.

**Methods:**

Fourteen clinicians from general practice, pediatrics, pediatric dermatology, pediatric allergy, and pediatric dietetics from UK and Ireland took part in an online modified Delphi study. Over three rounds, participants gave their anonymous opinions and received individualized and group feedback, based on the premise that all children had SPTs. The findings were discussed in an online workshop.

**Results:**

Of 18 symptoms, 12 were identified as relevant to immediate and 7 to delayed allergy. Regarding SPTs, there was consensus over which allergens to use for wheat and soy but not cow's milk or hen's egg; for all study foods, wheal size was determined as 0–1 mm negative, ≥ 5 mm sensitized, but between 2 and 4 mm, categorization varied by food. During the final workshop, consensus was reached on dietary advice for nine combinations of SPT results and dietary history.

**Conclusion:**

We attained consensus on how SPTs and dietary history for four common food allergens should be interpreted in young children under 2 years of age with AD. These pragmatic recommendations may support clinician education, consistency of decision‐making, and future research.

## 1 Introduction

Atopic eczema/dermatitis (AD) affects approximately 20% of children [1], most of whom have mild‐to‐moderate disease that can be managed with emollients and anti‐inflammatory treatments, usually topical corticosteroids (TCS). Topical therapies can be messy and time consuming, and parents commonly worry about their safety [2]. This combined with the perception that topical therapies only treat the symptoms and not the underlying cause, leads many caregivers to ask about food allergy (FA) tests to identify dietary triggers.

There is a recognized association between AD, food sensitization, and FA. Up to half of children with AD have a positive specific IgE or skin prick test (SPT) to at least one food with a significant proportion having clinical symptoms of FA [3]. The prevalence of FA in children with AD is highest aged 0–2 years (up to 39.2%) and is associated with early onset and more severe disease [4]. Caregivers of infants with AD commonly exclude foods without professional advice, in the belief that FA may be causing AD symptoms [5, 6]. However, excluding such foods, especially in infants with AD already sensitized to allergens, can lead to loss of tolerance with subsequent IgE‐mediated FA on accidental re‐exposure [7].

Tests for FA may be undertaken for different reasons, typically when symptoms suggest an immediate, IgE‐mediated FA. Some clinicians employ them when delayed, non‐IgE FA is suspected: when moderate/severe AD is difficult to control or when it is simply unclear from the clinical history whether FA contributes to AD symptoms [8]. Whatever the context for FA tests, including their inappropriate use, once performed clinicians need to advise parents on how to interpret them.

Given the variation in clinical practice and lack of evidence, we conducted a Delphi consensus exercise with UK clinicians to determine what dietary advice should be given to caregivers of children with AD who have SPT performed to four common food allergens.

## 2 Materials and Methods

### 2.1 Study Design

We undertook a modified version of the Delphi consensus method [9, 10], which is useful in areas of limited research, controversy, debate or lack of clarity. The exercise (between June and September 2022) comprised three anonymous survey rounds, each open a minimum of 2 weeks, and a final workshop.

The aim was to reach agreement on SPT results, thresholds and relevant allergy symptoms; and thereby what dietary advice to give to parents based on symptoms and SPT results for cow's milk, hen's egg, wheat and soy. These foods were chosen because they commonly cause FA in infants and young children in Europe [11], it can be difficult to determine from symptoms alone if their exclusion improves AD control, and they are being evaluated in the Trial of food allergy IgE tests eczema relief (TIGER) study (ISRCTN52892540).

### 2.2 Recruitment of Panel Members

Experts from five professional groups (allergists, dermatologists, dietitians, general practitioners (GPs) with an interest in allergy, and pediatricians) were approached through professional networks across UK and Ireland in May/June 2022. From 22 experts, 19 expressed an interest, with 14 able to participate. Participants were offered £50 for each survey and workshop, with an additional payment of £50 for engaging throughout. Panel members provided consent and data on personal/professional characteristics.

### 2.3 Consensus Process

We ran three rounds of surveys, completed online. All applied to children with mild or worse AD, under 2 years of age, with no prior diagnosis of FA to cow's milk, hen's egg, wheat or soy. Survey items related to the diet of the child and not of the breast‐feeding mother. Most survey items sought opinions by means of multiple choice rather than “agree” or “disagree” statements. Panelists were masked to other participants' survey answers. After every round, each panelist received an answers summary (own and panel aggregate) including items to be revisited. Comments were invited and used to modify or add survey items for subsequent rounds. After each round, items unchanged and with consensus (defined as agreement of 80% or above) were removed (Table S1).

Round one asked which symptoms (timing and type) should be considered as indicative of immediate and/or delayed FA; which SPT extracts to use; and how to interpret SPT results (Table S1). Following rounds asked respondents to choose between oral food challenge, food exclusion or food inclusion in various contexts (Table S1). This could include children without symptoms suggestive of FA, which differs from usual clinical practice where symptoms guide the use of tests.

Findings were summarized as a flowchart, which showed the most appropriate diagnostic outcomes depending on: ingested recently (yes/no/never); symptoms if ever ingested (none/immediate/delayed) and SPT results (negative/positive). We defined ingested recently as within the last 2 months. This was discussed at a 90‐min online workshop, chaired by an academic GP with an interest in dermatology, who was independent of the process.

### 2.4 Analysis

Data were exported from the online survey website and analyzed using Stata version 17.0 (StataCorp, LLC College Station, TX). Free‐text comments were collated under themes by L.G. and reviewed by the research team (L.G., M.J.R., R.B., R.M., I.S., and S.J.B.).

### 2.5 Measures to Prevent Bias

The project leads (L.G., primary care researcher from quantitative social sciences background, and M.J.R., academic GP with a research interest in skin and allergy) met monthly and reviewed responses with the steering group (all clinical academicians—R.B. pediatric consultant allergist, S.J.B. consultant dermatologist, and R.M. and I.S., dietitians).

None of the research team were members of the panel. Panelists were invited to be co‐authors, to credit their intellectual contribution and to represent diversity of opinion.

### 2.6 Ethics

The study was reviewed and approved by the University of Bristol Faculty of Health Science Research Ethics Committee (reference 10819).

### 2.7 Funding

This study was funded by a joint award from the Rosetrees Trust and The Stoneygate Trust (OoR2021\100007), both of which had no input into design, delivery, analysis, or reporting.

## 3 Results

### 3.1 Participation

The panel comprised 11 doctors (seven specialists and four GPs with an interest in allergy) and three pediatric dietitians (Table 1). Survey response rates were 100%, 93%, and 100% respectively. Two panelists were unable to attend the workshop.

**TABLE 1:** Panel characteristics.

Region of professional practice	Expertise					
	General practitioner with an interest in allergy	Pediatric allergist	Pediatric dietitian	Pediatric dermatologist	Pediatrician with an interest in allergy	Total (%)
England—London	—	1	3	—	—	4 (28.6)
England—South West and South East	1	1	—	—	1	3 (21.4)
England—East of England	1	—	—	—	1	2 (14.3)
England—Midlands	—	1	—	1	—	2 (14.3)
Outside of England (Jersey, Scotland, Rep Ireland)	2	—	—	1	—	3 (21.4)
Total (%)	4 (28.6)	3 (21.4)	3 (21.4)	2 (14.3)	2 (14.3)	14 (100.0)

### 3.2 Clinical Symptoms

Opinions were sought on 18 skin, gut, or airways symptoms. Consensus emerged (Table S2) for 12 immediate and 7 delayed allergy symptoms (Table 2).

**TABLE 2:** Immediate and delayed food allergy symptoms.

		Not had	How long after ingestion was the symptom noticed?	
			< 2 h	> 2 h
Skin symptoms				
i.	Rash, redness of skin	□	□	□
ii.	Hives (nettle rash)	□	□	□
iii.	Itching	□	□	□
iv.	Swelling around the face, lips or eyes	□	□	□
v.	Rough or bumpy, itchy skin	□	□	□
vi.	Worsening of eczema (new flare)	□	□	□
Gut symptoms				
vii.	Diarrhea	□	□	□
viii.	Constipation	□	□	□
ix.	Abdominal pain	□	□	□
x.	Blood or mucus in their stool	□	□	□
xi.	Vomiting	□	□	□
Airway symptoms				
xii.	Noisy or difficult breathing	□	□	□
xiii.	Wheezing	□	□	□
xiv.	Coughing	□	□	□
xv.	Sneezing	□	□	□
xvi.	Nasal congestion or constant runny nose	□	□	□
xvii.	Swelling of tongue or airway	□	□	□
xviii.	Hoarse voice or cry	□	□	□

*Note*: Symptoms shaded blue in < 2 h column = immediate; symptoms shaded green in > 2 h column = delayed.

### 3.3 SPT Allergens

The panel were asked to choose fresh foods or commercial extracts as reagent. Consensus was reached (Table 3) for commercial extracts for wheat (86%) and soy (83%). There was consensus for egg commercial extract (86%) but no further consensus between whole egg and egg white (Table 3). There was no consensus for cow's milk.

**TABLE 3:** Allergen reagent for skin prick tests: Source and proportion (%) of panelists in agreement.

Food	SPT reagent	
	Round one	Round two
Cow's milk	Commercial cow's milk extract (50%), Fresh cow's milk (50%)	Commercial cow's milk extract (58%)
Hen's egg	Commercial extract (86%) [whole egg or egg white]	Commercial whole egg extract (55%)
Wheat	Commercial wheat extract (86%)	—
Soya	Commercial soy extract (64%)	Commercial soy extract (83%)

### 3.4 SPT Thresholds

The panel were asked to interpret 0–9 mm SPT wheal sizes as negative, equivocal or positive. There was consensus for all study foods that 0–1 mm indicated a negative result, and 5 mm and above indicated a positive result (Table 4). There were uncertainties in the 2–4 mm range: no agreement at 3 mm for any food; 4 mm considered a positive result except for wheat; 2 mm considered a negative result for wheat and soy, but no agreement for cow's milk and hen's eggs.

**TABLE 4:** Interpretation of SPT thresholds as negative or positive over rounds one, two and three.

Food	Round		% panelists in agreement, by SPT wheal sizes (mm)									
			0	1	2	3	4	5	6	7	8	9
Cow's milk	1		92				85	85	92	92	100	100
	2			92								
	3				71	71						
Hen's egg	1		92				85	92	92	92	92	92
	2			92								
	3				64	71						
Wheat	1		92						85	85	92	92
	2			100	92			83				
	3					57	64					
Soya	1		92					85	85	92	92	92
	2			100								
	3				85	50	85					

*Note*: 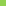 ≥ 80% agreement: SPT negative interpretation, 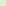 < 80% agreement: SPT negative or equivocal interpretation, 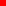 ≥ 80% agreement: SPT positive interpretation, and 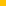 < 80% agreement: SPT positive interpretation.

### 3.5 Consensus on Decisions

**FIGURE 1:**
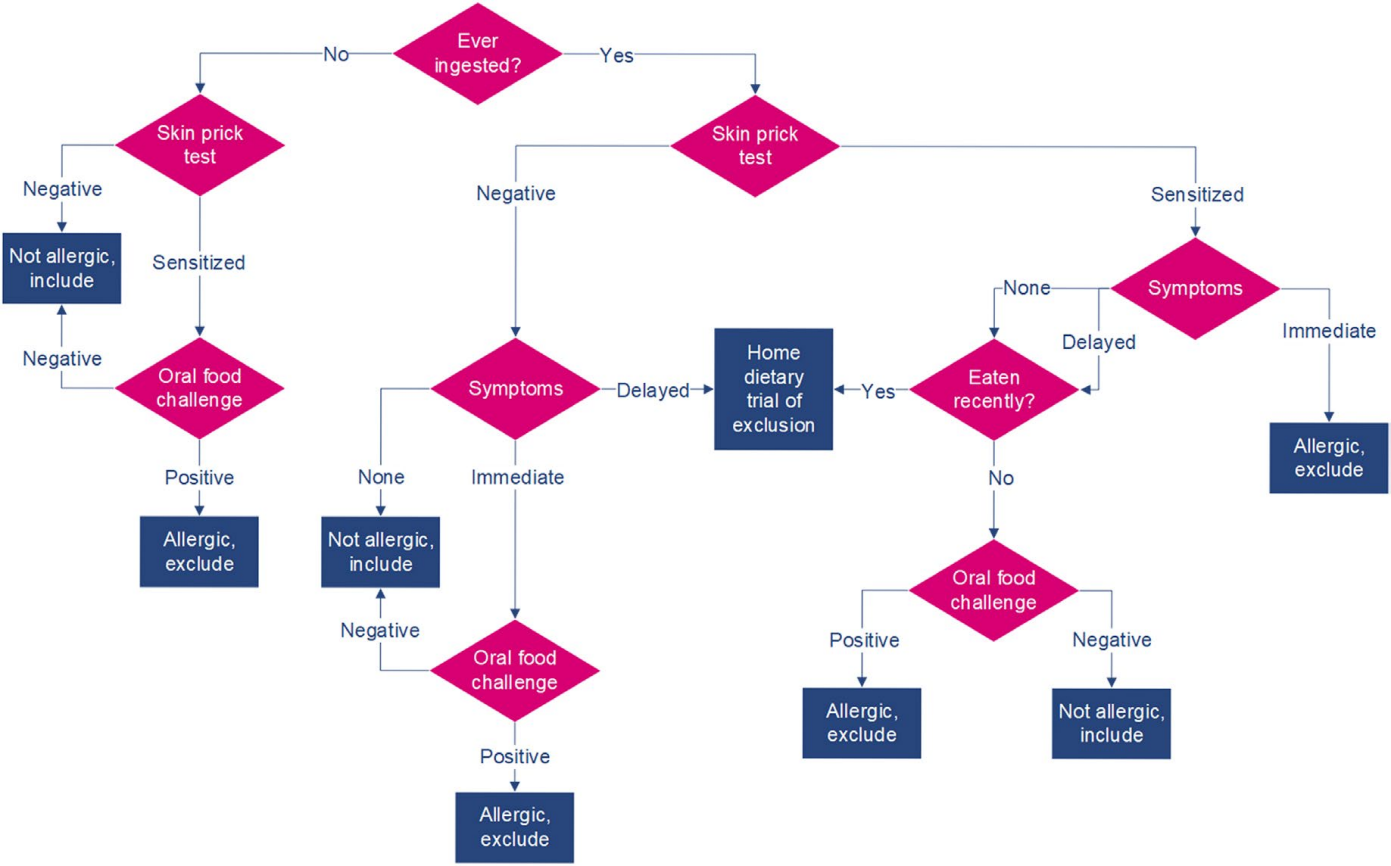
Dietary advice flowchart. **SPT**: negative < 3 mm, sensitized ≥ 3 mm. **Immediate symptoms**: < 2 h worsening of eczema (new flare), hives (nettle rash), itching, swelling around the lips, face or eyes, diarrhea, abdominal pain, vomiting, coughing, wheezing, hoarse voice or cry, swelling of tongue or airway/stridor or sneezing. **Delayed symptoms**: > 2 h worsening of eczema (new flare), itching, diarrhea, constipation, abdominal pain, blood and/or mucus in stool, vomiting. **Eaten recently**: ingestion within the last 2 months. **Advice**: *Not allergic, include *= continue to include in diet or reintroduce/introduce as normal if not currently/ever eaten. *Allergic, exclude* = exclude from diet until review in allergy clinic and/or oral food challenge. *Home dietary trial of exclusion* = exclude food for 4 weeks and reintroduce, to identify if any improvement/worsening in AD symptoms. Oral Food Challenge (OFC) = done under close medical supervision (in hospital) to find out if a child has an immediate allergic to a particular food. The child is gradually fed bigger doses of a single food, until the “top dose” is eaten without any symptoms, or a reaction occurs.

Agreement on dietary advice to be given, based on different combinations of symptoms and SPT results for the four different foods, evolved over rounds two and three (Table S3). The equivocal SPT category was removed and advice for children who have never ingested the food added. Outcomes were reviewed at the workshop (Figure S1), with all areas of prior disagreement resolved. Based on feedback, “positive” SPTs were relabelled as “sensitized.” The final flowchart (Figure 1) comprises nine pathways/outcomes.

## 4 Discussion

### 4.1 Summary

Through this exercise, a multi‐disciplinary panel of clinicians with expertise in pediatric AD/FA reached agreement on how to interpret SPTs to four foods in the context of immediate, delayed or no FA symptoms in a child less than 2 years of age, with AD. It is not an endorsement of the use of SPT in all cases of infantile AD, but rather an effort to clarify how to interpret results when such testing is done, including inappropriately.

Consensus was reached for the use of commercial extracts for wheat and soy but not which specific extracts to use for milk or egg SPT. There was also agreement on wheal sizes for definite negative and sensitized SPT results, but not in the 2–4 mm equivocal range. Finally, there was consensus on appropriate end points for combinations of symptoms and SPT results.

The resulting flowchart (Figure 1) can be used by clinicians to aid in the diagnosis and management of SPT results for FA in children with AD.

### 4.2 Strengths and Limitations

A strength of this exercise was the involvement of clinicians from different disciplines in generalist and specialist care. Engagement was high, frank views were shared and anonymity was retained until the workshop.

Making changes to the Delphi process might be seen as a limitation but such modifications are acceptable [12] and were necessary for the sake of brevity [13]. The online format facilitated commitment to the whole exercise and allowed the panel to be drawn from across the UK, reflecting regional practice variations. The short timescale may have helped recall of previous rounds.

This exercise was limited to four common food allergens. Peanut, for example, was not included because its presence in food is usually easier to determine. The findings may not apply to all children with AD and opinions outside the panel may vary. Persuasive arguments may have been made or received differently face‐to‐face. In consensus exercises, context is important [13]. The same questions presented in another setting may have elicited different responses.

Given the complexity of FA in children, it can be argued that decision‐making cannot be protocolized [14]. Taking an allergy‐focused history requires time and care, especially with severe AD of early onset, certain symptom complexes (e.g., co‐occurrence of skin and gut symptoms), when multiple foods are implicated, or prior FA diagnosis. Similarly, evidence of impact on the growth trajectory and/or micronutrient intake is an important consideration [15]. Clinicians will want to consider AD disease severity, known FAs, wheal size and positive predictive value of SPTs. While there was a lack of consensus with wheal sizes of 3–5 mm, ≥ 3 is often considered positive [16]. Oral food challenges might have to be delayed until a developmentally appropriate age. Delaying the introduction of, or excluding, allergenic foods may increase the risk of FA [17]. Therefore, benefits from routine FA testing to guide dietary advice for the management of AD must outweigh potential harms [18], and parents should still be supported to use topical therapies to treat their child's skin.

### 4.3 Findings in the Context of the Literature

Dietary modification by parents, without healthcare input, is common [6]. Delayed FA as the cause of mild AD symptoms or signs and the use of blood specific IgE or SPTs to guide dietary advice is controversial. Healthcare professionals working in the fields of allergy and pediatrics are more likely to request FA tests than those working in dermatology or general practice [8, 19].

A recent systematic review concluded that dietary elimination “may lead to a slight, potentially unimportant improvement in AD severity, pruritus, and sleeplessness” in patients with mild‐to‐moderate AD [18]. However, there were limited data on the psychosocial impact of food exclusion, developmental and nutritional deficits, and the risk of developing IgE‐mediated FA [17].

Algorithms exist to determine “when and how to evaluate for immediate (IgE‐mediated) FA in children with AD” [20] or to support the safe home introduction of tree nuts and peanut [21]. While a negative SPT may reassure parents, screening may be a distraction [22], and AD control with topical therapies may prevent egg allergy [23]. With early onset (< 6 months) AD, some clinicians advocate routine FA testing and elimination diets before complementary feeding starts [24].

Various forms of milk and egg SPT allergens are used in different clinics. Since conducting this Delphi study, new evidence supports commercial extract over fresh cow's milk [25].

### 4.4 Implications for Research, Policymakers, or Clinicians

Children with suspected FA in nonspecialist settings should, if possible, be promptly referred for further assessment because parental concerns about FA may become a barrier to effective use of topical treatments. Currently, the use of IgE FA tests in clinical settings should be confined to confirming or refuting diagnosis following an allergy‐focused history [19].

Although FA testing may be sought by parents to guide dietary exclusions, the findings from this exercise can also guide introduction or reintroduction of foods. The on‐going Trial of Food Allergy IgE Tests for Eczema Relief (TIGER) study [26] will provide definitive evidence about the value of the routine use of FA tests for AD control in young children.

## Author Contributions

M.J.R. conceived the idea and secured funding for the project with S.J.B. The project was led by M.J.R., delivered by L.G., with support from S.J.B., R.B., R.M., and I.S. M.J.R./S.J.B./R.B./R.M./I.S./L.G. recruited the panel. L.G./M.J.R. co‐led on data acquisition (survey design, piloting and administration), analysis and interpretation, and drafted the manuscript. S.J.B./R.B./R.M./I.S. contributed to survey design and piloting, data analysis and interpretation via discussions during regular research meetings. H.A., P.B., J.D., M.D., H.E.H., M.F., S.L., T.M., J.R., G.S., T.U., D.V., N.Y., and J.W. were members of the panel. All authors contributed to interpretation of the data and to critical revision of the manuscript for important intellectual content.

## Ethics Statement

The study was reviewed and approved by the University of Bristol Faculty of Health Science Research Ethics Committee (reference 10819). All panel members provided consent to participate in the Delphi exercise.

## Conflicts of Interest

J.W. has sat on clinical advisory panels to Allergy UK and Anaphylaxis UK. G.S. acts as Trustee to Allergy UK, contributed to an educational video on atopic dermatitis with Sanofi and is Principal Investigator for NARF study. R.M. is on the advisory panel of CoMISS supported by Nestle Health Science, has provided consultancy to Else Nutrition, and received honoraria for lectures from Nutricia/Danone, Abbott, and Reckitt Benckiser. I.S. has received honoraria for lectures from ThermoFisher, Royal College of GPs, TouchiME. M.J.R. is chief investigator on the TIGER (Trial of IgE food allergy tests for Eczema Relief) study (NIHR133464). S.J.B. has received research funding (but no personal financial benefits) from the Wellcome Trust (220875/Z/20/Z), UKRI, Medical Research Council, Rosetrees Trust, Stoneygates Trust, British Skin Foundation, Charles Wolfson Charitable Trust, anonymous donations from people with eczema, Unilever, Pfizer, Abbvie, Sosei‐Heptares, Janssen, European Lead Factory (multiple industry partners) and the BIOMAP consortium (EC‐IMI project ref. No 821511).

## Supporting information


Data S1.

Figure S1.

## Data Availability

The data that support the findings of this study are available on request from the corresponding author. The data are not publicly available due to privacy or ethical restrictions.
