# Supplementary material for: Food Allergy Test‐Guided Dietary Advice for Children With Atopic Dermatitis: A Consensus Study
Source: Pediatr Dermatol. 2024 Nov 11;42(2):259–66. doi: 10.1111/pde.15807 (PMC11950804; doi:10.1111/pde.15807)
Supplement: Supplementary file 1 — Data S1. [file PDE-42-259-s002.docx]

# Supplementary material

# Tables

Table S1: What questions or issues were asked or discussed when

|  | Round  one | Round  two | Round  three | Workshop | Post-workshop |
| --- | --- | --- | --- | --- | --- |
| Symptoms | ● | ○ |  | ○ | ○ |
| Allergens | ● | ○ |  |  |  |
| SPT thresholds | ● | ○ | ○ |  |  |
| Interpretation of findings |  | ● | ○ | ● |  |

Key: ● All items included
 ○ Some items included, e.g. where no consensus from previous stage

Table S2: Symptoms and timescales associated with an immediate (IgE)/delayed (non-IgE) type allergic reaction to food

|  | Round 1 | | | | | Round 2 | | | | Final | | |
| --- | --- | --- | --- | --- | --- | --- | --- | --- | --- | --- | --- | --- |
|  | IgE | | | Non-IgE | | IgE | Non-IgE | | | IgE | | Non-IgE |
| n | 14 | | | 14 | | (4) | (3) | | | 12 | | 7 |
| Skin symptoms | Rash, redness of skin | | | Rash, redness of skin | |  |  | | | Worsening of eczema (new flare) <2 hours | |  |
|  |  | | |  | |  | Rough or bumpy, itchy skin | | |  | |  |
|  |  | | |  | |  | New eczema flare | | |  | | Worsening of eczema (new flare) 2-24 hours |
|  | Hives (nettle rash) | | | Hives (nettle rash) | |  |  | | | Hives (nettle rash) < 2 hours | |  |
|  | Itching | | | Itching | |  |  | | | Itching < 2 hours | | Itching 2 hours to 2 days |
|  | Swelling around the lips, face or eyes | | | Swelling around the lips, face or eyes | |  |  | | | Swelling around the lips, face or eyes < 2 hours | |  |
| Gut symptoms | Diarrhoea | | | Diarrhoea | |  |  | | | Diarrhoea <24 hours | | Diarrhoea 2 hours to 2 days |
|  | Constipation | | | Constipation | |  |  | | |  | | Constipation 1-2 days |
|  | Abdominal pain/ bloating (incl. ‘colic’) | | | Abdominal pain/ bloating (incl. ‘colic’) | | Abdominal pain |  | | | Abdominal pain < 2 hours | | Abdominal pain 1-2 days |
|  | Blood and/or mucus in poo | | | Blood and/or mucus in poo | |  |  | | |  | | Blood and/or mucus in poo 1-2 days |
|  | Vomiting | | | Vomiting | |  |  | | | Vomiting <2 hours | | Vomiting 2 hours to 2 days |
| Airways symptoms | Coughing | | | Coughing | |  |  | | | Coughing <2 hours | |  |
|  | Wheezing | | | Wheezing | |  |  | | | Wheezing <2 hours | |  |
|  | Noisy or difficulty breathing | | | Noisy or difficulty breathing | | Hoarse voice or cry |  | | | Hoarse voice or cry <2 hours | |  |
|  |  | | |  | | Swelling of tongue or airway/ stridor |  | | | Swelling of tongue or airway/stridor <2 hours | |  |
|  | Sneezing | | | Sneezing | |  |  | | | Sneezing <2 hours | |  |
|  | Nasal congestion or runny nose | | | Nasal congestion or runny nose | | Nasal congestion or runny nose | Nasal congestion or runny nose | | |  | |  |
|  | |  |  | |  |  | |  |  | |  |  |
| Key: | |  | Consensus – include | |  | Not consensus | |  | Consensus – exclude | |  |  |

Table S3: Scenarios where panel reached over 80% agreement on dietary advice to be given

|  |  | **Negative SPT result** | | | | **Sensitised STP result** | | | |
| --- | --- | --- | --- | --- | --- | --- | --- | --- | --- |
| **Exposure** | **Reaction** | Cow’s milk | Hen’s egg | Wheat | Soya | Cow’s milk | Hen’s egg | Wheat | Soya |
| Recent | None | ● | ● | ● | ● |  |  |  |  |
|  | Immediate | ● | ○ | ● | ○ | ● | ● | ● | ● |
|  | Delayed | ● | ● | ● | ● |  |  |  |  |
| Not recent | None | ● | ● | ● | ● | ● | ○ |  | ○ |
|  | Immediate | ● |  | ● | ● | ● | ● |  |  |
|  | Delayed | ● | ● | ● | ● | ○ |  |  | ○ |
| None | n.a. |  |  | ○ |  | ○ | ○ | ○ | ○ |

Key: ● Consensus at Round 2
 ○ Consensus at Round 3

# Figure


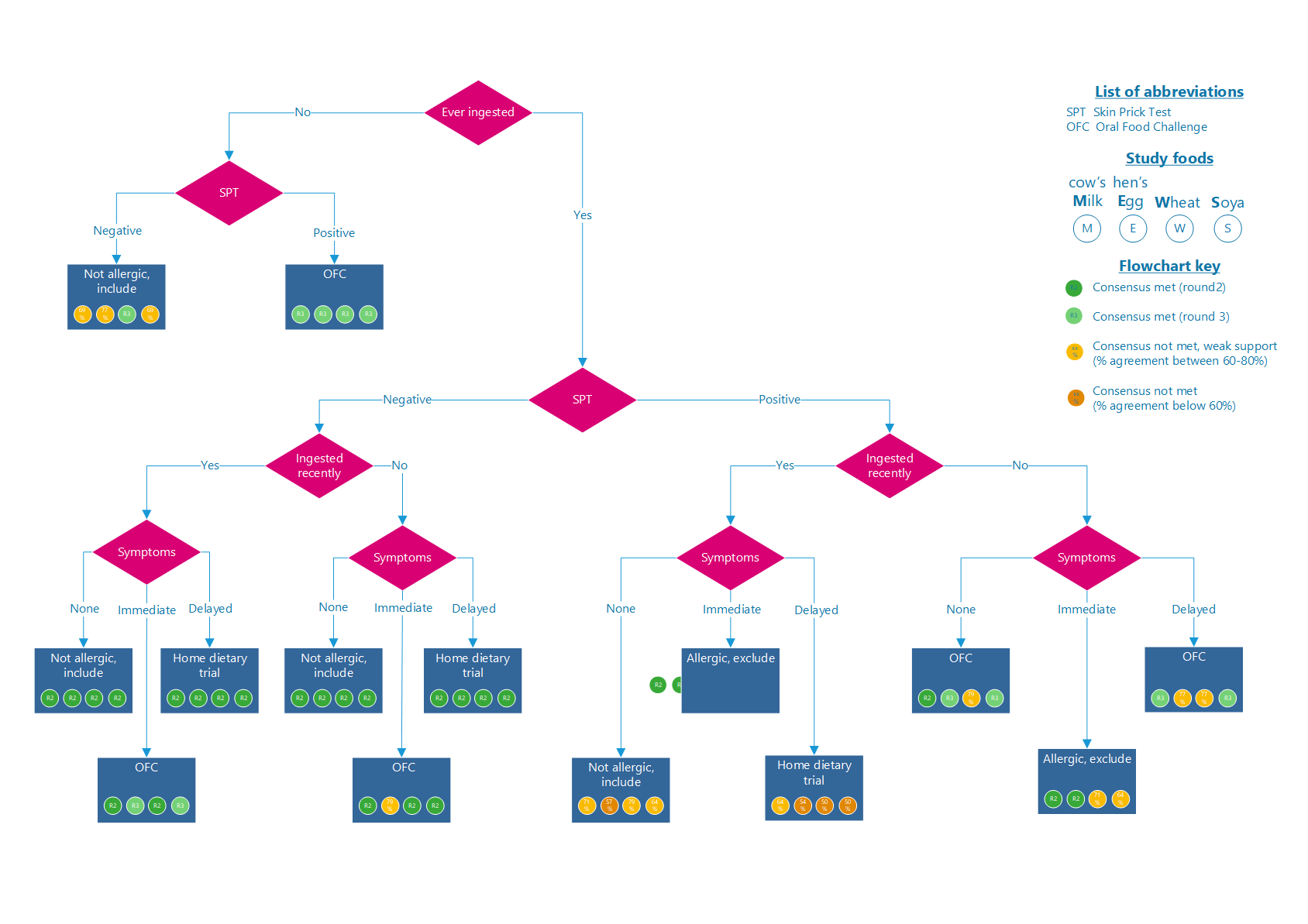


Figure S1: Results from rounds 2 and 3 on dietary advice

Footnotes

**SPT**: negative <3 mm, positive >= 3 mm.

**Immediate symptoms**: <2 hours. Worsening of eczema (new flare), hives (nettle rash), itching, swelling around the lips, face or eyes, diarrhoea, abdominal pain, vomiting, coughing, wheezing, hoarse voice or cry, swelling of tongue or airway/stridor or sneezing.

**Delayed symptoms**: >2 hours. Worsening of eczema (new flare), itching, diarrhoea, constipation, abdominal pain, blood and/or mucus in poo, vomiting.

**Ingested recently**: ingestion within the last 2 months.

**Advice**: *Not allergic, include* = continue to include in diet or reintroduce/introduce as normal if not currently/ever eaten; *Allergic, exclude* = exclude from diet until review in allergy clinic and/or Oral Food Challenge; *Home dietary trial of exclusion* = exclude food for four weeks and reintroduce, to identify if any improvement/worsening in AD symptoms. *Oral Food Challenge (OFC)* = done under close medical supervision (in hospital) to find out if a child has an immediate allergic to a particular food. The child is gradually fed bigger doses of a single food, until the “top dose” is eaten without any symptoms, or a reaction occurs.
