# Supplementary figures and images for: Food Allergy Test‐Guided Dietary Advice for Children With Atopic Dermatitis: A Consensus Study
Source: Pediatr Dermatol. 2024 Nov 11;42(2):259–66. doi: 10.1111/pde.15807 (PMC11950804; doi:10.1111/pde.15807)

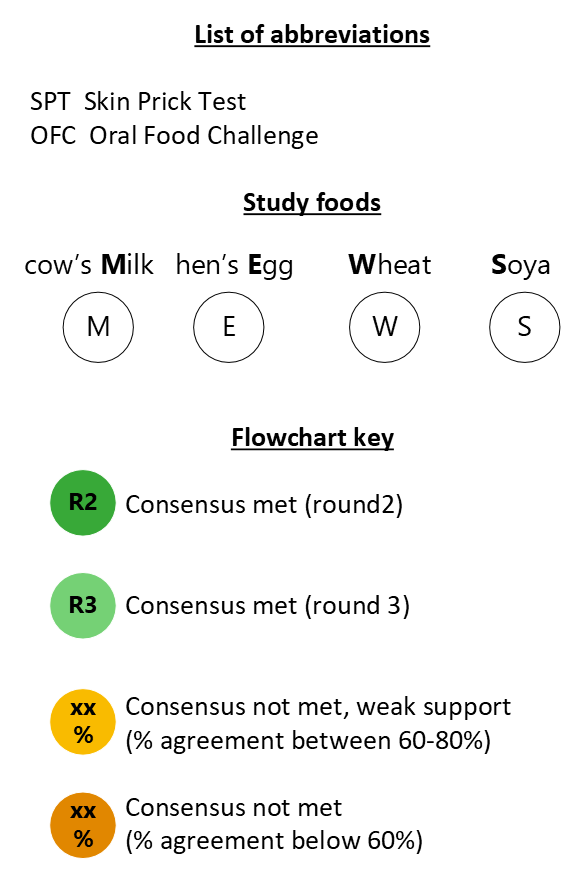

Supplement: Supplementary file 2 — Figure S1. [file PDE-42-259-s001.zip › Figure_S1b.png]

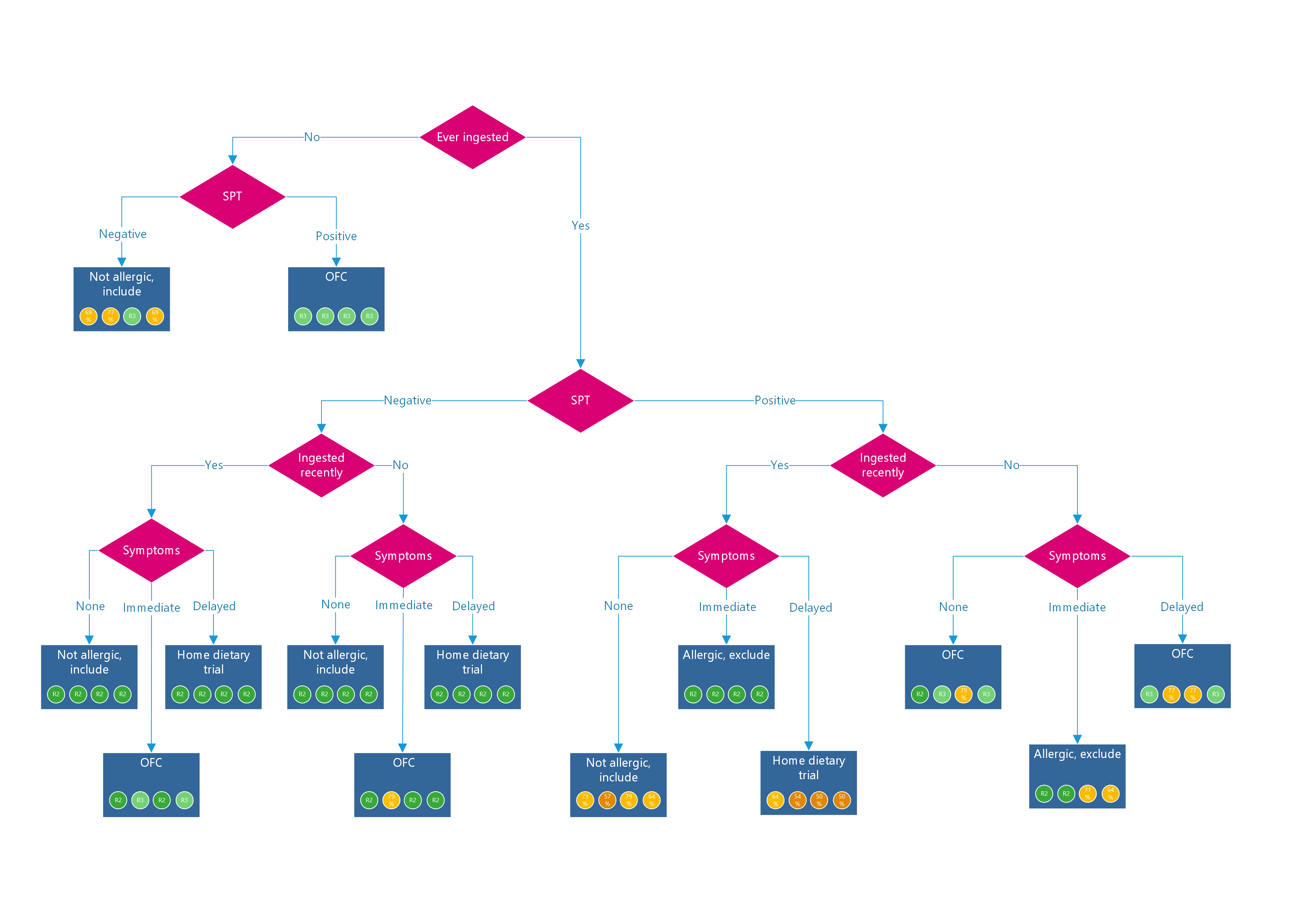

Supplement: Supplementary file 2 — Figure S1. [file PDE-42-259-s001.zip › Figure_S1a.png]
